# Neutralizing Antibody Therapeutics for COVID-19

**DOI:** 10.3390/v13040628

**Published:** 2021-04-07

**Authors:** Aeron C. Hurt, Adam K. Wheatley

**Affiliations:** 1 F. Hoffmann-La Roche Ltd., 4070 Basel, Switzerland; 2 Department of Microbiology and Immunology, University of Melbourne at the Peter Doherty Institute for Infection and Immunity, Melbourne, VIC 3000, Australia; a.wheatley@unimelb.edu.au

**Keywords:** SARS-CoV-2, COVID-19, neutralizing antibody, resistance, casirivimab, imdevimab, bamlanivimab, etesevimab

## Abstract

The emergence of SARS-CoV-2 and subsequent COVID-19 pandemic has resulted in a significant global public health burden, leading to an urgent need for effective therapeutic strategies. In this article, we review the role of SARS-CoV-2 neutralizing antibodies (nAbs) in the clinical management of COVID-19 and provide an overview of recent randomized controlled trial data evaluating nAbs in the ambulatory, hospitalized and prophylaxis settings. Two nAb cocktails (casirivimab/imdevimab and bamlanivimab/etesevimab) and one nAb monotherapy (bamlanivimab) have been granted Emergency Use Authorization by the US Food and Drug Administration for the treatment of ambulatory patients who have a high risk of progressing to severe disease, and the European Medicines Agency has similarly recommended both cocktails and bamlanivimab monotherapy for use in COVID-19 patients who do not require supplemental oxygen and who are at high risk of progressing to severe COVID-19. Efficacy of nAbs in hospitalized patients with COVID-19 has been varied, potentially highlighting the challenges of antiviral treatment in patients who have already progressed to severe disease. However, early data suggest a promising prophylactic role for nAbs in providing effective COVID-19 protection. We also review the risk of treatment-emergent antiviral resistant “escape” mutants and strategies to minimize their occurrence, discuss the susceptibility of newly emerging SARS-COV-2 variants to nAbs, as well as explore administration challenges and ways to improve patient access.

## 1. Introduction

In late 2019, the novel betacoronavirus Severe Acute Respiratory Syndrome Coronavirus 2 (SARS-CoV-2) emerged from an animal reservoir into humans, causing a respiratory infection known as Coronavirus Disease 2019 (COVID-19) [1]. The high transmissibility and rapid global spread of SARS-CoV-2 has led to a worldwide COVID-19 pandemic that has continued for more than a year, leading to a huge burden on healthcare and society [2]. Although the majority of COVID-19 cases are asymptomatic or involve mild-to-moderate symptoms, up to 10% of patients can develop severe disease resulting in hospitalization and death [3]. During the first year of the pandemic, around 95 million cases and 2 million deaths were attributed to COVID-19 [4], leading to an urgent need for effective treatment strategies to limit adverse outcomes.

At the start of the pandemic, no evidence-based treatments were available for COVID-19 despite the emergence and global spread of the related SARS-CoV-1 (Severe Acute Respiratory Syndrome [SARS]) in 2003, and continued ongoing sporadic infections with another related coronavirus, Middle East Respiratory Syndrome (MERS), that first arose in 2012 [5]. Thus, initial studies involved a multi-pronged therapeutic approach, whereby several treatment options were investigated, including repurposed broad-spectrum antivirals (e.g., remdesivir and lopinavir/ritonavir), anti-parasitic drugs (e.g., hydroxychloroquine and ivermectin) and immunomodulators (e.g., dexamethasone and tocilizumab), which were found to have varying degrees of efficacy [6,7,8,9,10,11,12,13,14,15].

Convalescent plasma therapy showed initial promise in the treatment of COVID-19 patients [16], but phase 3 clinical trial data have generally proved disappointing [17,18], likely due to variation in the active concentration of neutralizing antibodies (nAbs) and subsequent lack of standardized doses between patients. Pharmaceutical-grade monoclonal nAbs circumvent these limitations and have emerged as the first SARS-CoV-2–specific treatments to become available [16,19,20,21]. Only a small number of nAbs have previously been approved and used for the clinical management of viral diseases, including palivizumab for Respiratory Syncytial Virus prophylaxis [22], and, more recently, treatments for Ebolavirus disease, including a single nAb monotherapy (ansuvimab-zykl) [23] and a triple monoclonal nAb cocktail (REGN-EB3) [24,25]. Although several nAb candidates have been developed for influenza, none have advanced beyond clinical trials as a result of underwhelming efficacy [26].

This article reviews the nAbs currently in development for COVID-19. The concept of viral neutralization is complex and wide-ranging, and our article defines neutralization as a reduction in viral infectivity caused by binding of antibodies to the surface of viral particles to prevent viral replication [27]. We provide an overview of the efficacy of nAbs for treatment and prophylaxis in different settings. In addition, the challenges of drug resistance and approaches to improve the access and availability of nAbs in the future is discussed.

## 2. SARS-CoV-2 nAb Development

SARS-CoV-2 enters host cells through interactions between the viral spike protein and the cellular angiotensin-converting enzyme 2 (ACE2) receptor [28]. The spike protein is therefore a rational target for leading COVID-19 vaccines and nAb-based therapies [29,30]. The spike protein is a homo-trimer, with each monomer composed of two subunits (S1 and S2). S1 contains the N-terminal domain (NTD) and the receptor binding domain (RBD), which mediates the ACE2 interaction, while S2 mediates membrane fusion. Immune recognition of the spike protein surface is constrained via a “glycan shield” of complex carbohydrates [31]. However, the comparative accessibility of the RBD and its critical role in facilitating viral entry make this domain the major target for protective antibodies elicited by vaccination, and the dominant target of nAbs identified to date. Although the RBD is a major target of nAbs, neutralizing non-RBD epitopes have also been identified, including nAbs that bind the NTD in S1 [32,33] and others that bind the S2 subunit [34], which could offer additional possibilities for novel nAb combination approaches in the future.

A range of approaches has been utilized to generate or identify nAbs to SARS-CoV-2. Arguably the most powerful approach has been screening convalescent patient peripheral blood mononuclear cells to enrich and identify individual B cells with potent SARS-CoV-2 binding affinity [33,35,36,37,38,39]. Additional approaches to identify potent nAbs have included screening of naïve human fragment antigen-binding (Fab) phage display libraries [40] and the use of genetically humanized mice (VelocImmune^®^) [41], which can be immunized against SARS-CoV-2, and the resulting fully human, high potency nAbs screened and purified [42]. Hundreds of nAbs have been identified through these approaches and screened using in vitro and in vivo methods to identify the best candidates for clinical development. Animal studies have shown that nAb treatment can reduce viral replication in the respiratory tract of rhesus macaque SARS-CoV-2 challenge models, as well as reduce pathology in hamster COVID-19 models [37,43,44]. Beyond treatment, nAbs have also demonstrated a prophylactic effect in animals exposed to SARS-CoV-2, providing protection in some high-dose settings, whilst driving a reduction in viral loads that suggests some potential to reduce onward virus transmission [37,43,44].

SARS-CoV-2 nAbs may be modified in the fragment crystallizable (Fc) region to improve pharmacological properties [45,46]. For example, extending nAb half-life might be desirable in the prophylaxis setting, to maximize the duration of protection offered by a single dose, thus limiting the need for supplemental administrations. In vivo half-life of immunoglobulin G (IgG) is modulated by interactions with cells bearing the neonatal Fc receptor (FcRn), and several mutations of the Fc region have been identified that increase affinity to FcRn to extend antibody half-life [46]. Several SARS-CoV-2 nAbs have undergone such modifications, including the fully recombinant human nAb, VIR-7831, which is based on an antibody from a convalescent patient, but engineered to have an extended half-life and enhanced lung bioavailability [47]. AZD7442 is another example and is a cocktail of two nAbs where each has been engineered in the Fc domain to both extend half-life and limit interactions with cellular Fc receptors, thereby minimizing the potential for antibody-dependent enhancement (ADE) [48].

Other modifications to minimize the risk of ADE via unwanted effector functions (e.g., proinflammatory cytokine secretion and complement activation) have also been made, such as the introduction of double leucine to alanine mutations at position 234 and 235 (“LALA mutants”) in the Fc portion of the nAb etesevimab (LY-CoV016) and MW05 [45,46,49,50].

## 3. Evaluation of SARS-CoV-2 nAbs in the Treatment and Prophylaxis Settings

**Figure 1:**
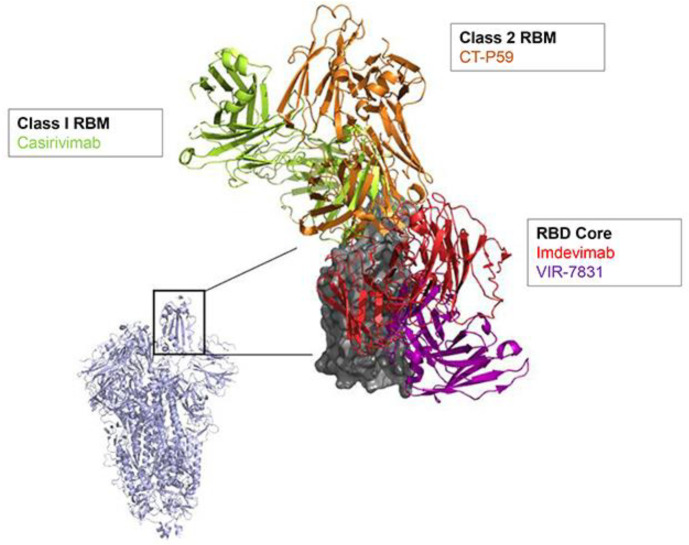
Localization of SARS-CoV-2 nAbs in late-stage clinical development. The distribution of RBD regions recognized by casirivimab (PDB: 6XDG), CT-P59 (PDB: 7CM4), imdevimab (PDB: 6XDG) and VIR-7831 (S309) (PDB: 6WPS) are compiled and illustrated on the SARS-CoV-2 virus (PDB:6W41). Epitopes are clustered proximal to RBM bound by ACE2, or alternatively at the distal RBD core. SARS-CoV-2 RBD is depicted in gray; the inset shows its location in the context of the entire spike protein (depicted in lavender). Only those nAbs with publicly available structures are shown. RBD, receptor binding domain; RBM, receptor binding motif.

The breadth of promising pre-clinical evidence has so far resulted in more than 20 nAbs being evaluated in various clinical trial settings [51]. Figure 1 illustrates the target epitopes in the RBD for nAbs in late-stage clinical development where structures are publicly available. Having a range of therapeutics targeting different viral epitopes is desirable as it increases the options, should some fail to maintain efficacy against new variant viruses (see later section on resistance). Of the nAbs in clinical development, several have progressed to phase 2 or 3 trials evaluating the treatment of ambulatory and/or hospitalized COVID-19 patients, as well as in the prophylaxis setting. An overview of ongoing clinical trials (Phase 2 and 3) is provided in Table 1.

### 3.1. Efficacy of Treatment for Ambulatory Patients

Several nAbs are currently being investigated for the treatment of ambulatory patients with COVID-19. However, detailed efficacy data are available only for the single nAb bamlanivimab (LY-CoV555) and the cocktail of bamlanivimab/etesevimab being developed by Eli Lilly and AbCellera, and the nAb cocktail of casirivimab and imdevimab (previously REGN10933 and REGN10987, respectively) being developed by Regeneron and F. Hoffmann-La Roche Ltd. (Table 1).

For bamlanivimab, which binds the RBD of the spike protein [16], an ambulatory phase 2/3 trial known as BLAZE-1 (NCT04427501) found that the virological effect among 452 randomized patients was mixed, with a significant reduction in mean viral load only being observed in the “middle” dose group receiving 2800 mg (difference −0.53, 95% CI −0.98, −0.08; *p* = 0.02), but not in the lower dose group (700 mg) (difference −0.20, 95% CI −0.66, 0.25; *p* = 0.38), or most perplexingly in the highest dose group (7000 mg) (difference 0.09, 95% CI −0.37, 0.55; *p* = 0.70) (doses were fixed and not weight-based) [52]. The reasons for these differences are not understood, but could involve the “prozone effect,” whereby high concentrations of antibodies can impair the formation of immune complexes. Bamlanivimab resulted in fewer patients requiring hospitalization or emergency department visits (1.0% in the 700 mg dose group, 1.9% in the 2800 mg dose group, and 2.0% in the 7000 mg dose group) compared with 6.3% in placebo-treated patients [52]. Based predominantly on this reduction in the need for subsequent health resource utilization, the 700 mg dose of bamlanivimab was granted Emergency Use Authorization (EUA) by the US Food and Drug Administration (FDA) for treatment of ambulatory patients at high risk of progressing to severe COVID-19 (including hospitalization) [53]. The BLAZE-1 study is also evaluating the combination of bamlanivimab with etesevimab in ambulatory COVID-19 patients. Virological data from 577 patients showed additional potency derived from the cocktail of bamlanivimab/etesevimab, which was associated with a significant reduction in log_10_ viral load (−0.57) versus placebo (*p* = 0.01), while bamlanivimab monotherapy did not result in significant reductions [54]. Compared with placebo, the cocktail was also shown to reduce the number of hospitalizations (2.1% vs. 7.0%; risk reduction 70%, *p* = 0.0004) and deaths (0 vs. 10) among 1035 patients [55]. This led to the FDA granting an EUA for the cocktail in the treatment of mild to moderate COVID-19 in patients aged ≥12 years with COVID-19 and a high risk of progression to severe disease [56]. Similarly, the European Medicines Agency (EMA) has issued advice that the bamlanivimab/etesevimab cocktail can be used to treat COVID-19 patients who do not require supplemental oxygen and who are at high risk of progressing to severe disease [57]. The EMA advice also noted that bamlanivimab monotherapy can be considered as a treatment option in this patient population, despite uncertainties around the benefits of monotherapy.

**Figure 2:**
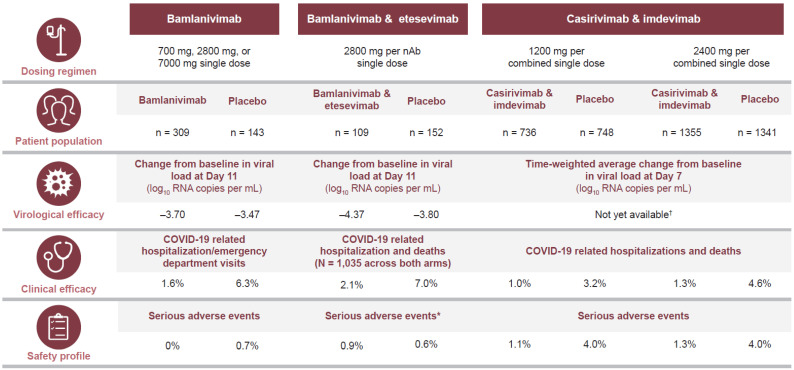
Comparison of the Key Clinical, Virological and Safety Data for Neutralizing Antibodies with EUA for Treatment of Ambulatory Patients with COVID-19. * Based on 112 patients (combination arm) and 156 patients (placebo arm). ^†^ Interim data from the phase 1/2 portion of the trial reported a time-weighted average change from baseline of −1.74 log_10_ RNA copies per mL in casirivimab/imdevimab-treated patients (n = 143; combined results from 2400 mg and 8000 mg dose arms) versus a −1.34 log_10_ RNA copies per mL change from baseline in the placebo arm (n = 78).

The nAbs casirivimab and imdevimab, being developed by Regeneron and F. Hoffmann-La Roche Ltd., bind non-overlapping sections of the RBD [42] and are being investigated as a cocktail in the ambulatory setting in a phase 1/2/3 trial (NCT04425629) taking place across several countries. Data have been reported from the phase 3 study (N = 4567) evaluating 1200 mg or 2400 mg casirivimab/imdevimab versus placebo in patients with ≥1 risk factor for severe COVID-19. The trial met its primary endpoint and revealed that the casirivimab/imdevimab cocktail significantly reduced the risk of hospitalization or death by 70% in the 1200 mg dose arm (*p* = 0.0024) and 71% in the 2400 mg dose arm (*p* < 0.0001) compared with placebo [58]. All key secondary endpoints were also met, including a four-day reduction in the median duration of symptoms (both doses; *p* < 0.0001) versus placebo. At the time of writing, detailed virological data have not been reported from the phase 3 trial. However, interim data from the first 275 patients (phase 1/2 portion) showed that the casirivimab/imdevimab cocktail demonstrated virological efficacy resulting in an overall reduction in viral load of 0.25 log_10_ RNA copies/mL (95% CI −0.60, 0.10) for the 2400 mg dose and a reduction of 0.56 log_10_ RNA copies/mL (95% CI −0.91, −0.21) for an 8000 mg dose (combined dose reduction was 0.41 log_10_ RNA copies/mL, 95% CI −0.71, −0.10) versus placebo at Day 7 [59]. No data on infectious virus titers or time to cessation of viral shedding endpoints have been reported, similar to the situation with bamlanivimab or indeed any nAb study to date. An ongoing dose-ranging phase 2 companion trial in low-risk symptomatic or asymptomatic non-hospitalized patients with COVID-19 (NCT04666441) showed significant and comparable viral load reductions across casirivimab/imdevimab doses ranging from 300 mg to 2400 mg delivered via intravenous (IV) or sub-cutaneous (SC) routes [58]. The casirivimab/imdevimab cocktail has received EUA by the US FDA for the treatment of ambulatory patients with mild to moderate COVID-19 and a high risk of hospitalization [60], and the EMA has similarly recommended casirivimab/imdevimab for use in COVID-19 patients who do not require supplemental oxygen and who are at high risk of progressing to severe COVID-19 [61]. The EMA has also started a rolling review of the data for bamlanivimab/etesevimab [62], casirivimab/imdevimab [63], and CT-P59 [64]. Figure 2 provides a side-by-side comparison of the key clinical, virological and safety data of current treatments with FDA EUA (bamlanivimab monotherapy, bamlanivimab/etesevimab cocktail and casirivimab/imdevimab cocktail) for ambulatory COVID-19 patients.

Other nAbs are undergoing evaluation in phase 2/3 placebo-controlled studies in ambulatory patients with COVID-19, including VIR-7831 alone (NCT04545060) or in combination with bamlanivimab as part of the BLAZE-4 trial [65].

### 3.2. Efficacy of Treatment for Hospitalized Patients

Hospitalized patients with COVID-19 are a difficult-to-treat population that are associated with extremely poor outcomes, including ~25% mortality and significant requirements for critical care [66,67]. The casirivimab/imdevimab cocktail is being investigated in a placebo-controlled phase 1/2/3 trial of hospitalized adult patients with COVID-19 (NCT04426695). However, following an independent data monitoring committee evaluation, a recommendation was made to proceed with recruitment only in patients requiring no or low-flow oxygen, as the benefit–risk profile for patients requiring high-flow oxygen or mechanical ventilation was considered unfavorable [68]. This trial is ongoing, but a futility analysis was conducted among 217 patients requiring low-flow oxygen who were SARS-CoV-2 seronegative at baseline, a group that were shown to have an increased risk of death compared to those that had already mounted an antibody response. In these patients, casirivimab/imdevimab treatment was found to reduce viral load by 0.54 log_10_ RNA copies/mL at Day 7 and 0.63 log_10_ RNA copies/mL at Day 11 and lower the risk of death or receiving mechanical ventilation (hazard ratio: 0.78; 80% CI 0.51, 1.2) compared with placebo-treated patients [69]. Although the reduction in the risk of death was not statistically significant, the trend was sufficient to continue with the trial, and it is expected that the full data set alongside the ongoing RECOVERY trial (NCT04381936), which has so far recruited >5000 patients to receive casirivimab/imdevimab or placebo plus standard of care (SoC), will allow rigorous evaluation of the efficacy of the nAb cocktail in this group of hospitalized patients.

Several other nAbs, including bamlanivimab, have been and are being evaluated in hospitalized patients with COVID-19 as part of the phase 3 ACTIV-3 trial, which is sponsored by the National Institute of Allergy and Infectious Diseases (NCT04501978). The master protocol has an adaptive design that permits investigational agents plus SoC (remdesivir, unless contraindicated) to be compared against placebo plus SoC. Preliminary results showed that bamlanivimab failed to provide clinical benefit in hospitalized COVID-19 patients, as it did not expedite clinical improvement nor hospital discharge compared with placebo [70].

Trials investigating VIR-7831 and the BRII-196/BRII-198 cocktail as part of the ACTIV-3 design have recently begun, where each sub-study will aim to recruit an additional 700 patients on top of those enrolled to determine initial safety and efficacy outcomes [71]. Additional studies in hospitalized patients with COVID-19 are expected, including a phase 2/3 study with the humanized recombinant nAb SCTA01 (NCT04644185).

### 3.3. Efficacy of Prophylaxis

Vaccines will remain the most appropriate measure to deliver COVID-19 protection to the majority of individuals. However, it is likely that nAbs will play an important role in providing short-term protection for those who are unvaccinated or do not respond well to vaccination (e.g., immunocompromised individuals), as well as during periods when vaccines do not provide sufficient protection from circulating variant viruses (e.g., during periods of vaccine mismatch). Several nAbs are being evaluated for prevention of infection/symptomatic disease, with initial data from nursing homes and households demonstrating their beneficial role in post-exposure prophylaxis.

Bamlanivimab has been investigated for post-exposure prophylaxis efficacy in a phase 3 trial involving 299 residents and 666 staff within nursing homes (NCT04497987). After 8 weeks of follow-up, bamlanivimab prophylaxis was found to reduce the likelihood of symptomatic COVID-19 by 57% (odds ratio 0.43, *p* = 0.00021), and this increased to 80% in a pre-specified group of residents (odds ratio 0.20, *p* = 0.00026) [72]. Of the 299 residents enrolled in the study, four deaths occurred that were attributed to COVID-19, all of which were in the placebo arm.

SC injection of the casirivimab/imdevimab cocktail is also being investigated for post-exposure prophylaxis in an ongoing placebo-controlled phase 3 trial involving healthy adult household contacts of individuals with a positive SARS-CoV-2 reverse-transcription polymerase chain reaction (RT-PCR) test (NCT04452318). Initial results involving an exploratory analysis of the first ~400 participants showed that casirivimab/imdevimab prophylaxis resulted in complete prevention of symptomatic COVID-19 (0/186) among household contacts, compared with a frequency of 3.6% (8/223) among household contacts receiving placebo, and a 51% reduction in PCR-positive infection (5.4% [10/186] vs. 10.3% [23/223]) [73]. Although ten asymptomatic household contacts receiving the cocktail were found to be PCR-positive, duration of viral RNA detection in these individuals (who remained asymptomatic) did not exceed one week and none had viral loads >10^4^ RNA copies/mL. By comparison, viral RNA was detectable for several weeks in PCR-positive individuals in the placebo arm, with 62% having viral loads >10^4^ RNA copies/mL [73].

Phase 3 trials examining the AZD7442 cocktail are ongoing in both the pre- (NCT04625725) and post-exposure (NCT04625972) prophylaxis settings. AZD7442 is a combination of two nAbs derived from convalescent serum that demonstrated effective SARS-CoV-2 prophylaxis in a rhesus macaque model [74]. Both antibodies have been engineered to have an extended half-life estimated to offer 6–12 months of protection following a single administration, as well as reduced Fc binding to minimize the risk of ADE [48]. An overview of ongoing prophylaxis studies is provided in Table 2.

## 4. Susceptibility to Currently Circulating Variants

As the key viral entry protein, the SARS-CoV-2 spike is uniquely exposed to selective pressure from host antibodies, which can drive rapid antigenic drift and evolution. Therefore, viruses may develop resistance to nAbs either through the natural evolution of the virus (i.e., due to selection pressure exerted by the host immune system in the absence of drug treatment) or resistance can arise in an individual patient due to the selection pressure of treatment during viral replication [75]. In the latter case, such variants are usually of minimal concern because acute respiratory infections are typically cleared in immunocompetent individuals relatively quickly, but in the former case, if escape mutations are present in circulating viruses the utility of that treatment may be substantially reduced [75].

Strategies to minimize the risk of escape mutants include targeting highly conserved regions of the viral spike, or utilizing a cocktail of nAbs rather than monotherapy. As conserved epitopes tend to overlap with regions essential for viral function, mutations in this region would be more likely to compromise viral fitness, and consequently be less likely to become fixed in the viral population [20]. This approach has been adopted in the case of VIR-7831, which was derived from a SARS convalescent patient and binds to an epitope that is conserved between SARS-CoV-1 and SARS-CoV-2 [16]. The cocktail approach, where multiple nAbs bind distinct epitopes, has been demonstrated to minimize the de novo generation of escape mutants during in vitro passaging experiments, compared with the rapid selection of resistant variants under pressure from a single nAb [44]. This approach is being utilized in several instances for the treatment or prophylaxis of COVID-19, e.g., casirivimab/imdevimab, bamlanivimab/etesevimab and the AZD7442 cocktail.

Current knowledge of mutations leading to nAb resistance remains limited, particularly for bamlanivimab. In the BLAZE-1 study where bamlanivimab treatment was investigated in ambulatory COVID-19 patients, SARS-CoV-2 escape mutants were reported in ~6% of patients treated with placebo, increasing to ~10% in patients receiving the highest dose of bamlanivimab [52]; however, the authors did not report which mutations were present in these viruses. The detection of resistant viruses in patients in the placebo arm suggests these were naturally circulating, raising concerns regarding the future prevalence of these viruses, and secondly that an additional ~4% of patients receiving the nAb developed resistance under treatment.

In an in vitro study, the casirivimab/imdevimab cocktail successfully neutralized SARS-CoV-2 spike variants containing mutations identified from publicly available sequence data, as well as escape mutants generated under selection pressure from either casirivimab or imdevimab alone [76]. Notably, the casirivimab/imdevimab cocktail did not select for resistance mutants in this study [76]. However, a recent study that mapped the effect of single-residue mutations of all amino acids in the RBD on the binding of spike protein to casirivimab/imdevimab identified one mutation (E406W) that significantly reduces inhibition by this cocktail; notably, E406W cannot be generated by a single-nucleotide change and has not been reported in any sequence databases to date [77]. Nonetheless, this study highlights mutations that, if present in circulating strains, could escape either casirivimab, imdevimab or etesevimab and will likely be an important resource for assessing future viral variants observed by surveillance efforts. In the ambulatory study of the casirivimab/imdevimab cocktail (NCT04425629), the G446V mutation was detected in three out of 66 (4.5%) patients with sequencing data available, including two patients at baseline, suggesting G446V is a circulating resistance mutant potentially arising due to selection pressure from host antibodies [78]. The G446V mutation showed a 135-fold reduction in susceptibility to imdevimab relative to wild-type virus, but retained susceptibility to the casirivimab/imdevimab cocktail [78]. Currently, there are insufficient data to know whether certain nAbs or nAb cocktails will have more or less propensity towards selection for resistance than others.

Significant attention has been paid in recent months to the emergence of variant SARS-CoV-2 viruses with key mutations in the spike, particularly those that have emerged first in the UK (B.1.1.7), South Africa (B.1.351) and Brazil (P1/B.1.1.28). A recent study found that the so called “South African” virus variants (B.1.351) are resistant to bamlanivimab due to the E484K spike substitution. Of additional concern is that even as a cocktail, bamlanivimab and etesevimab are predicted to have no activity against this variant due to the latter nAb having highly reduced activity due to the K417N substitution that is also present in this variant [77,79,80].

The E484K and K417N substitutions in the B.1.351 strains are also responsible for reduced inhibition by casirivimab, but because the other nAb in the cocktail (imdevimab) remains unaffected by these mutations, the overall neutralization achieved by the cocktail was reduced by only 5–20-fold, depending on whether the actual variant virus or a pseudovirus was used. [79]. However, the clinical effect corresponding to a 5–20-fold reduction based on in vitro neutralization is unknown. A summary of emerging resistance mutations on nAbs with EUA from naturally occurring and engineered viruses is provided in Table 3. As our current understanding of nAb resistance mutations is far from comprehensive (Table 3), continued surveillance of emerging SARS-CoV-2 variants and their susceptibility to nAbs and vaccines should remain a priority.

## 5. Approaches to Improve nAb Access and Availability

Besides the challenges associated with cost/access, large-scale manufacturing and storage [81], the largest hurdle that currently exists, in both the ambulatory outpatient and prophylaxis settings, is mode of administration. Bamlanivimab and the bamlanivimab/etesevimab and casirivimab/imdevimab cocktails have been granted EUA by the FDA for treatment of COVID-19 in the outpatient setting, but are administered by IV infusion, a procedure that, whilst commonplace in hospitals, creates substantial challenges in a community setting. Infusion centers, pop-up sites or in-home visits are being considered and may be safer from a public health perspective (reducing the risk of potentially spreading SARS-CoV-2 in the community) while offering more convenience to the patient. In an attempt to simplify the administration of bamlanivimab and bamlanivimab/etesevimab, the FDA have approved shortened IV infusion times from 60 min to 16 min and 21 min, respectively, which is expected to help ease the burden in healthcare settings [82]. An alternative to IV infusion for nAb delivery includes SC injection, which is being investigated for the casirivimab/imdevimab cocktail in the COVID-19 post-exposure prophylaxis setting (NCT04452318) [73], and nasal sprays, such as the nAb EU126-M2, which has shown pre-clinical prophylactic efficacy by blocking SARS-CoV-2 infection in mice for up to seven days after virus exposure [83]. Adeno-associated virus vectors may also offer opportunities for delivering nAb-expressing gene therapy constructs, an approach that has shown promise in pre-clinical mouse and ferret influenza models [84]; limiting expression to respiratory epithelial cells may be safer than using widely-expressed constructs. Clearly, any drug that is orally available would offer huge advantages in the outpatient setting, and developing novel technologies to make nAbs orally available is a major goal of ongoing research activities [81].

The approval of several efficacious COVID-19 vaccines is an integral part of national COVID-19 public health plans, and a thorough understanding of the potential interactions and implications of delivering vaccines after recent infusion with a nAb is warranted. Although no data exist in this regard, the US Centers for Disease Control and Prevention have recommended that vaccination should be deferred for ≥90 days in patients who have received nAbs. This recommendation appears to be based on the potential interference of nAbs in mounting an effective vaccine-induced immune response [85].

## 6. The Role of nAbs in Preparing for Future Novel Coronavirus Pandemics

SARS-CoV-2 is the third pathogenic novel coronavirus to infect humans within the last 20 years, suggesting there is a high likelihood of other novel coronavirus pandemics in the coming decades [19]. Treatments with broad activity against coronaviridae will be important to utilize in the initial response phase of a future coronavirus pandemic. Existing nAbs with broad activity may have utility in this regard, for example VIR-7831, which was isolated from a convalescent SARS patient, targets conserved epitopes on viral spike proteins and has activity across SARS-CoV-1 and SARS-CoV-2 [16]. In addition, development of new broadly reactive nAbs with potential to be evaluated up to phase 1 safety trials could be completed during interpandemic periods, allowing the stockpile of an arsenal of therapeutics that can be rapidly manufactured and evaluated when new viruses emerge. Indeed, the National Institutes of Health recently suggested that stockpiling of nAbs with broad activity should be part of broader pandemic preparedness planning for coronavirus and for other emerging pathogens of concern [86].

## 7. Conclusions

Since the beginning of the COVID-19 pandemic, there has been a massive global effort to harness the potential of nAbs for inhibiting the SARS-CoV-2 virus. There are hundreds of nAbs in pre-clinical development for COVID-19 treatment and prophylaxis, and several promising candidates have progressed to evaluation in the clinical trial setting. Emerging data suggest nAbs are particularly effective in preventing patients with risk factors from progressing to severe disease requiring hospitalization. Studies in hospitalized patients are ongoing, and whilst nAbs offer strong potential in this setting, the late timing of treatment onset means the effective window of antiviral effect may be limited. As the clinical data continue to emerge, it is likely that we will see several options for COVID-19 treatment and prophylaxis becoming available, and it will be important to ensure challenges associated with IV administration in the outpatient setting are met to ensure the best possible patient access. The potential for nAb resistant variants to circulate is an ongoing concern; therefore, continued surveillance of circulating viruses and detailed knowledge of the relationship between spike mutations and effectiveness of nAbs will be important to ensure treatment guidelines remain relevant. In this respect, future studies investigating the safety and efficacy of different combinations of nAbs will be important, as well as combination of nAbs with direct-acting antivirals, which may provide an even higher barrier to treatment-emergent resistance.

## Figures and Tables

**Table 1:** Overview of Phase 2 or 3 Clinical Trials Evaluating Neutralizing Antibodies for COVID-19 Treatment or Prophylaxis.

nAb	Sponsor	Monotherapy or Cocktail	Administration Route	nAb Source	NCT Number	Phase
Ambulatory patients						
BGB-DXP593	BeiGene	Monotherapy	IV infusion	Convalescent plasma	NCT04551898	Phase 2
MW33	Mabwell (Shanghai) Bioscience Co., Ltd.	Monotherapy	Unknown	Recombinant	NCT04627584	Phase 2
Bamlanivimab ^1^	AbCellera/Eli Lilly and Company	Monotherapy	IV infusion	Convalescent plasma	NCT04518410	Phase 2/3
CT-P59	Celltrion	Monotherapy	IV infusion	Convalescent plasma	NCT04602000	Phase 2/3
VIR-7831	Vir Biotechnology, Inc. GlaxoSmithKline	Monotherapy	IV infusion	Convalescent plasma	NCT04545060	Phase 2/3
Bamlanivimab & etesevimab	AbCellera/Eli Lilly and Company	Cocktail	IV infusion	Convalescent plasma/recombinant	NCT04427501	Phase 3
Casirivimab & imdevimab ^1^	Regeneron/F. Hoffmann-La Roche Ltd.	Cocktail	IV infusion	Convalescent plasma/humanized mice	NCT04425629	Phase 1/2/3
Hospitalized patients						
Casirivimab & imdevimab	Regeneron/F. Hoffmann-La Roche Ltd.	Cocktail	IV infusion	Convalescent plasma/humanized mice	NCT04426695	Phase 1/2/3
SCTA01	Sinocelltech Ltd.	Monotherapy	IV infusion	Recombinant	NCT04644185	Phase 2/3
VIR-7831	Vir Biotechnology, Inc. GlaxoSmithKline	Monotherapy	IV infusion	Convalescent plasma	NCT04501978	Phase 3
BRII-196 & BRII-198	Brii Biosciences	Cocktail	IV infusion	Convalescent plasma	NCT04501978	Phase 3
TY027	Tychan Pte. Ltd.	Monotherapy	IV infusion and SC injection	Engineered	NCT04649515	Phase 3
Bamlanivimab	AbCellera/Eli Lilly and Company	Monotherapy	IV infusion	Convalescent plasma	NCT04501978	Phase 3
Prophylaxis						
AZD7442 (combination of AZD8895 & AZD1061)	AstraZeneca	Cocktail	SC injection	Convalescent plasma	NCT04625725 NCT04625972	Phase 3 Phase 3
Bamlanivimab & etesevimab	AbCellera/Eli Lilly and Company	Cocktail	IV infusion	Convalescent plasma/recombinant	NCT04497987	Phase 3
Casirivimab & imdevimab	Regeneron/F. Hoffmann-La Roche Ltd.	Cocktail	SC injection	Convalescent plasma/humanized mice	NCT04452318	Phase 3

^1^ Emergency Use Authorization granted by US Food and Drug Administration to treat outpatients at high risk of severe disease; IV, intravenous; nAb, neutralizing antibody; SC, subcutaneous.

**Table 2:** Overview of Ongoing Phase 3 Prophylaxis Studies.

NCT Number	Comparison	Target Recruitment (N)	Primary Efficacy Outcome	Study Population
NCT04452318	Casirivimab & imdevimab vs. placebo (post-exposure prophylaxis)	2450	Proportion of participants with RT-PCR-confirmed SARS-CoV-2	Asymptomatic healthy contacts exposed to a household member with a SARS-CoV-2 infection, with no history of prior SARS-CoV-2 infection
NCT04497987	Bamlanivimab ± etesevimab vs. placebo (post-exposure prophylaxis)	5000	Percentage of participants with COVID-19 within 21 days of detection	Nursing home residents or staff with a positive SARS-CoV-2 test and no prior history of SARS-CoV-2 infection or receipt of a SARS-CoV-2-specific vaccine or monoclonal antibodies
NCT04625725	AZD7442 vs. placebo (pre-exposure prophylaxis)	5000	Incidence of SARS-CoV-2 RT-PCR positive symptomatic illness	Healthy individuals with no prior history of a positive SARS-CoV-2 diagnosis nor previous receipt of a SARS-CoV-2-specific vaccine or monoclonal antibodies
NCT04625972	AZD7442 vs. placebo (post-exposure prophylaxis)	1125	Incidence of SARS-CoV-2 RT-PCR positive symptomatic illness	Healthy contacts with potential exposure to an individual with a SARS-CoV-2 infection, with no prior history of a positive SARS-CoV-2 diagnosis nor previous receipt of a SARS-CoV-2-specific vaccine or monoclonal antibodies

RT-PCR, reverse transcription polymerase chain reaction.

**Table 3:** Spike Amino Acid Substitutions in SARS-CoV-2 That Reduce Binding of nAbs with Food and Drug Administration Emergency Use Authorization.

nAb	S1 Mutations [77,79]
Bamlanivimab	E484K
Etesivimab	K417N, A457V, N460T, E484K
Casirivimab	E406W, K417E/N, Y453F, L455Y, E484K, F486I/K/V, Y489H, Q493K
Imdevimab	242–244del, E406W, N439K, N440D, K444Q, V445A

The table is not intended to be comprehensive; it is based on a very limited number of studies and there is a need for additional data in this area.
